# Could social interaction reduce the disposition effect? Evidence from retail investors in a directed social trading network

**DOI:** 10.1371/journal.pone.0246759

**Published:** 2021-02-11

**Authors:** Xuejun Jin, Rui Li, Yu Zhu

**Affiliations:** 1 Department of Finance, School of Economics, Zhejiang University, Hangzhou, China; 2 Department of Operations and Information Systems, David Eccles School of Business, University of Utah, Salt Lake City, Utah, United States of America; BeiHang University School of Economics and Management, CHINA

## Abstract

With data collected from a directed social trading network, this paper investigates how social interaction affects the disposition effect. We constantly observe a negative association between them: After being exposed to social interaction, a trader’s odds ratio to sell a paper gain stock decreases by 9% to 10%, depending on different model settings. We then test the mechanisms of social interaction by decomposing it into three channels: learning intensity (willingness to learn), learning quality (information advantage through learning), and public scrutinization (exposure of trading outcome to others). We find that all three channels contribute to a smaller disposition effect. Specifically, our findings support the claim that public scrutinization promotes self-consciousness and reduces disposition effect. Also, our results extend previous studies on investors’ information advantage by suggesting that it could also help to mitigate the disposition effect through the reduction of uncertainty. Overall, this paper suggests a positive role of social trading platforms in helping investors make better decisions.

## Introduction

The disposition effect is usually considered as an investment mistake: It describes the behavioral bias that investors sell wining assets too quickly while holding on to losers for too long [1]. Traditional explanations for the disposition effects often rely on personal preferences (e.g., Prospect Theory [1]) or personal attributes (e.g., investment experience [2, 3], education and learning ability [4]). But as retail investors are increasingly relying on social media to collect information and form investment decisions, interpersonal communication starts to play a more critical role in shaping investors’ behavior. However, how social interaction affects the disposition effect is much less discussed.

The lack of empirical studies on the interplay of social interaction and the disposition effect could be partially attributed to the limitations of data: For such a study, researchers need to collect both trading records and social network data for the same group of investors, but traditional data sources such as social media [5, 6] or brokerage data [7] could only provide parts of it. In this paper, we solve the problem by introducing a new data source, the “social trading platform,” where both trading records and social interaction are observable.

A social trading platform is an online platform that allows investors to make investment decisions by observing or even directly copying others’ trading records. It has two primary user groups: the signal providers who publish trading signals by showing their transaction records, and the signal followers who determine whether to leverage the incoming signals [8]. In a sense, a social trading platform resembles a mutual fund but with a critical difference: The transactions record of signal providers are fully transparent, meaning they are accessible to everyone with no delay, while for mutual funds, only a part of their position is disclosed, plus a regular one-month reporting lag.

A stream of studies that use social trading platform data to investigate the disposition effect have emerged, where they attribute the disposition effect to factors like peer pressure [9, 10], social-image [11, 12], and self-conscious [13, 14]. However, the conclusions are divergent. For example, [9, 10, 12, 15] explains social interaction as a way of public scrutinization and finds that it strengthens the disposition effect because investors want to preserve their reputation by delaying recognizing losses when their transactions are being exposed to others. On the other hand, [13, 14] argues that social interaction promotes self-consciousness and helps investors correct mistakes more quickly, which reduces the disposition effect.

Several shortcomings in extant literature may explain the disagreeing conclusions. First, the social network structure in some studies is undirected, where two traders must both give consent before they could establish a connection (like Facebook). In such an undirected (mutual) network, it is challenging to distinguish effect from peers and effect from oneself but is later reflected on the peers. This situation is identified as the “reflection problem” [16, 17] because it is like one standing in front of a mirror: It is difficult to tell if it is the person that drives the image in the mirror or the other way around.

Second, some researches use signal providers’ transaction data, resulting in a biased representation of real-world retail investors. Unlike signal followers, whose primary goal is investment return, signal providers usually have additional monetary incentives to attract followers [8]. They may be overly risk-taking or overly cautious. In this way, these signal providers act more like fund managers, not retail investors.

Third, most studies test the effect of social learning by splitting the data into two periods: the period without social interaction (the control group) and the period with social interaction (the treatment group). While it is an effective and clear approach, it cannot reveal the different channels through which social interaction takes effect, because the only change between the two groups is the presence of social interaction. To have a deeper understanding of the social mechanism, we need to identify its channels.

To solve the above problems, we collect data from Xueqiu.com (xueqiu literally means “snowball”), China’s largest social trading platform, and develop new measures of the social interaction channels. Unlike the undirected network in some studies [9, 18], the network of Xueqiu is directed, meaning there is a distinctive difference between “following someone” and “being followed.” Like Twitter, the forming of a link between two users in Xueqiu does not require mutual consent. Such a directed network reduces the reflection problem because it is now possible to identify effects from the fans to the followed and the impact from the followed to the fans.

More importantly, although traders on Xueqiu can publish their signals and copy from others’ signals as in a typical social trading platform, they cannot get monetary rewards by attracting followers. Xueqiu works more like a mutual help club, where club members share investment ideas by publishing their transactions for others’ reference. Because of the lack of monetary incentives for attracting followers, traders on Xueqiu are less biased and may represent the real-world retail investors better [14].

We investigate the effects of social interaction using two methods. First, following [9], we split the data into periods with and without social interaction, and then compute the change in the disposition effect between the two periods. The split of periods is based on the following observation: We find that most of the new users of Xueqiu will make a few trades before they start to follow someone. In this paper, we name the period stating from one’s joining of the network to her first following as the “pre-follow” stage (works like the control group), and the rest as the “post-follow” stage (works like the treatment group). Trades made during the post-follow stage are more likely to be influenced by social interaction. By computing the disposition effect of these two periods, we show that social interaction helps to reduce the disposition effect.

However, such a discrete splitting approach treats social interaction as a whole, hence cannot reveal the channels through which social interaction affects the disposition effect. How to measure social interaction continuously and also show its channels? To this end, we introduce three variables: learning intensity, learning quality, and public scrutinization, each representing one channel that has been identified in the literature. Public scrutinization, essentially the number of followers, measures the influence of the peers. Previous studies [9–15] have shown that public scrutiny could strengthen self-image while maintaining behavior or promote self-consciousness, resulting in an increase or reduction in the disposition effect. By contrast, learning intensity and learning quality measure the effect originated from the focal investor: how her active observation and learning from the peers impact her disposition effect. We define learning intensity as the number of followings. A large learning intensity suggests a strong motivation to establish connections with others, hence a stronger willingness to learn from peers. We then define learning quality as the average centrality of a trader’s neighbors, capturing her information advantage through learning from the network [19]. This paper finds that all three channels help reduce the disposition effect, supporting [13, 14].

Taken together, our results have three important implications in the stream of literature on social interaction, disposition effect, and social trading platforms. First, whether social interaction increases or reduces the disposition effect does not have an agreement in literature. This disagreement is partially due to the limitation of data. By collecting transaction records and social interaction from a directed network, this paper mitigates the reflection problem and supports the conclusion that social interaction reduces the disposition effect [13, 14].

Furthermore, this paper proposes a new method to decompose the effect of social interaction into three channels: learning intensity (willingness to learn), learning quality (information advantage through learning), and public scrutinization (exposure of trading outcome to others). Extant literature does not distinguish these channels and treats social interaction as a whole. By breaking down the social mechanism into these channels, we can better test theories that lead to the same direction of change. We show that all three channels help to reduce the disposition effect.

Third, this paper contributes to the stream of literature on social trading networks. Social trading networks are innovative and valuable data source that can simultaneously provide data about inter-personal interaction and trading records. Previous research topics that utilize social trading network data include the studies of interplay of social learning and trust [10, 20, 21], transparency [11, 13], and investment outcome [22, 23]. In this paper, we further exploit the value of social trading network by using it to investigate how social interactions contribute to investors’ behavioral biases.

The remainder of this paper is organized as follows. Section “Data” describes the data and descriptive statistics. Section “Methods” presents the two approaches that we use to examine the effects of social learning. Section “Results and discussion” presents the empirical results of how social learning influences the disposition effect. Section “Conclusion” concludes this paper.

## Data

### Xueqiu as a social trading platform

Here we introduce Xueqiu’s social trading system and how it differs from other social trading platforms. Like all social trading platforms, Xueqiu allows its users to publish their trades and follow/copy from each other. The core concept of Xueqiu’s social trading system is the portfolio. A portfolio is like an independent fund: Its transactions are recorded, and its return is calculated and published by the platform. Portfolios can be followed without the creator’s consent. Once followed, the latest trades made to the portfolio will be pushed to the follower’s desktop or mobile devices.

A trader can create two types of portfolios: the virtual and the real (money). A virtual portfolio does not involve any real-money transaction and is mainly used to test or demonstrate investment strategies. By contrast, a real portfolio, is directly linked to the trader’s brokerage account and reflects the trader’s actual gain or loss. One could create as many as 20 virtual portfolios, but only one real portfolio is allowed.

Virtual and real portfolios have different accessibility. Virtual portfolios are public, meaning anyone can see its trading history and follow it. Real portfolios, however, work as an exclusive mutual help club: By becoming a real portfolio creator, a trader must agree to share her brokerage account transactions with other real portfolio creators, but in return, the trader is granted access to other real portfolios’ transaction history. For those traders who only create virtual portfolios, real portfolios are invisible to them. This mutual help club design differs from other social trading platforms, where publishing transaction records and attracting followers could earn monetary rewards.

**Fig 1:**
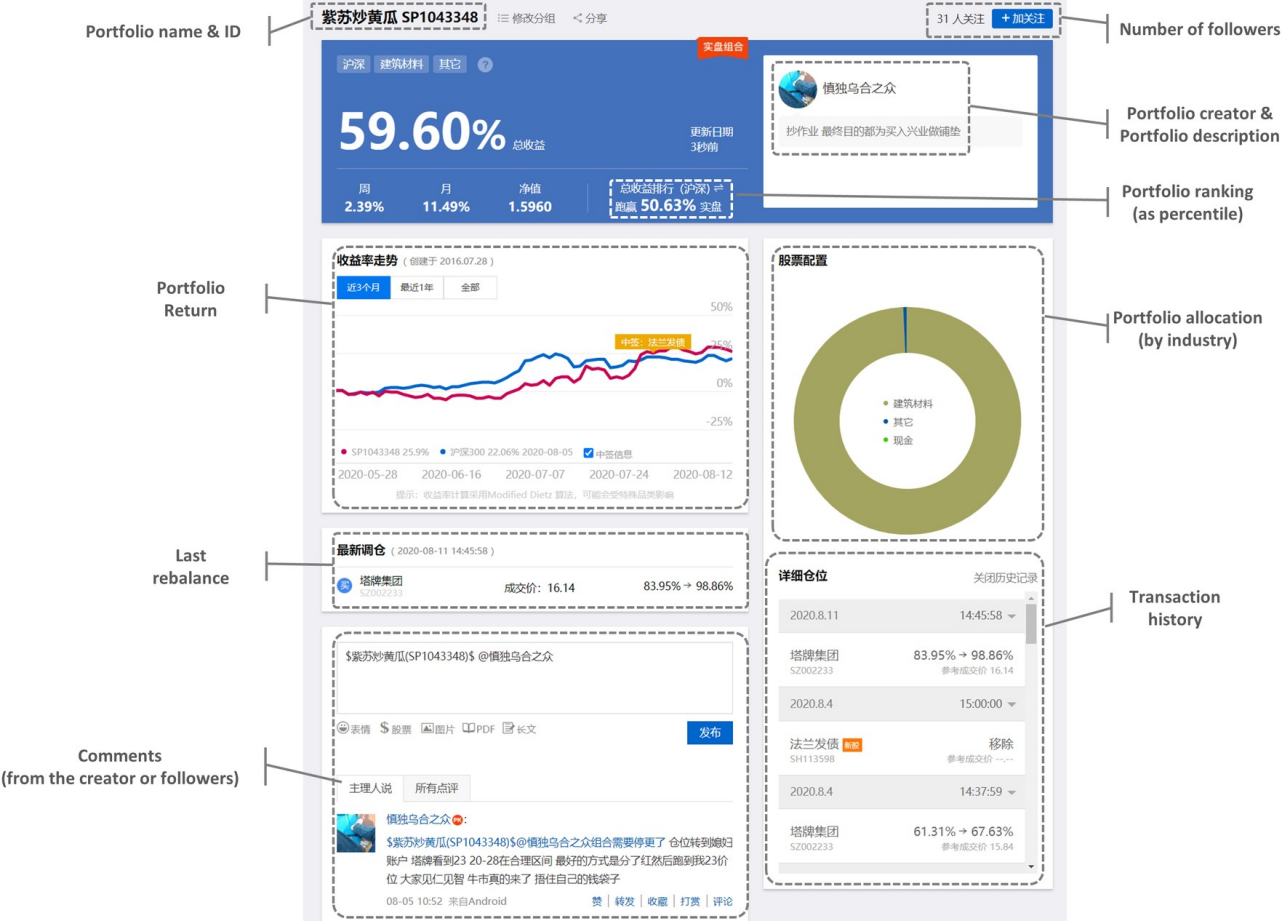
Profile page of a real portfolio.

In summary, the benefit of becoming a real portfolio creator is knowing what others are trading, while the cost is that the trader’s own transactions are also subject to scrutinization by others. Fig 1 shows a real portfolio’s profile page. The page of a virtual portfolio is very similar.

We collect two groups of data from Xueqiu: the transaction data and the social network data. For transaction data, we collect all stock trades made by real portfolio owners from June 2016 to March 2018. As explained above, real portfolio owners can see others’ real portfolios. Therefore, we registered as a real portfolio owner and crawled the transaction history webpages of other real portfolio owners. There are 601,087 trades executed by 4,802 traders. Because one trader can only create one real portfolio, the number of traders also equals the number of real portfolios. We clean the collected data by two criteria. First, invalid transaction records, such as trade with negative trading volume, are removed. Second, portfolios with empty records or were active less than one day are further removed. This filtering procedure reduces the data to 4,732 traders/portfolios with 541,774 trades, of which 211,315 are buy trades. Short selling is not allowed.

The transaction data is used to determine the gain/loss status of a trader’s position, which will be used in computing the disposition effect. We track each trader’s stock-level position and recompute its average cost by the end of the trading day. A stock position is denoted as a paper loss (gain) at day *t* if it is unsold and the closing price of that day is lower (higher) than its average cost. Similarly, if a stock is sold and the selling price is lower (higher) than the average cost, we denote it as a realized loss (realized gain).

**Fig 2:**
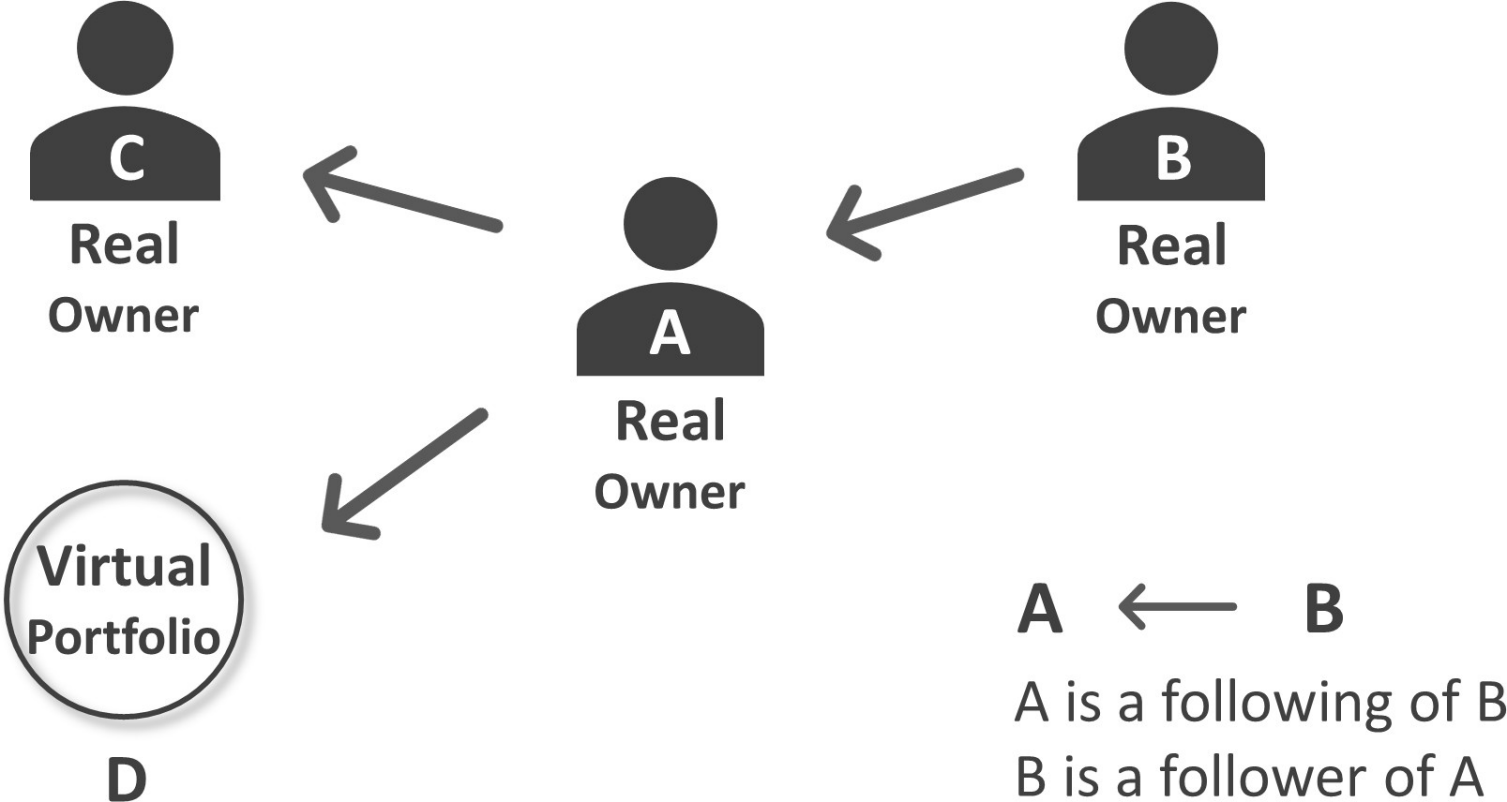
Followings and followers. This figure shows the definition of a following and a follower. A, B, C represent the real portfolio owners (and the real portfolios created by them) in our sample. D represents a virtual portfolio. For trader A, we say B is her followers and C and D are her followings.

The second group of data collected from Xueqiu is the social network (the followings and followers) of the 4,732 real portfolio owners. Fig 2 illustrates the definition of a following and a follower. Assume A, B, C are the traders in our sample. Since one trader can only create one real portfolio, A, B, and C also represent these traders’ real portfolios. We say B is a follower of A, or A is a following of B, if B adds A’s portfolio into her watchlist so that B will be notified whenever A makes a new trade. In Fig 2, link A → C or link B → A represents such connection. However, the following target of a real portfolio owner does not need to be another real portfolio: She can also follow a virtual portfolio, such as link A → D. In addition, because a trader’s social network will grow with time, we collect the number followings and followers at the end of every trading day.

### Summary statistics

Table 1 shows the summary statistics. In Table 1(A), we present statistics about traders’ transactions. We define portfolio life as the number of days between its creation date and the day when it makes the last trade in our sample. On average, a portfolio lasts for around one year and makes a bit more than 110 trades. We also find traders have a strong tendency to avoiding realizing losses: The average number of sales at a loss is 10.6, which is only one-third of that at a gain (30.6). Table 1(A) also shows that, on average, a trader only holds 2.75 stocks simultaneously and has traded about 22 stocks during the entire sample period. When it comes to investment outcomes, Table 1(A) shows that Xueqiu users yield a mild 3.7% median return, with a few yielding extreme negative and positive ones.

**Table 1:** Summary statistics.

	Min	Mean	Median	Max	S.D.
A. Transactions					
Portfolio life (days)	0	348.8	372	638	176.1
Number of trades per trader	1	114.5	117	200	68.1
Number of sales per trader	0	41.2	39	104	28.1
Number of sales at a gain per trader	0	30.6	28	94	21.4
Number of sales at a loss per trader	0	10.6	8	68	9.86
Number of daily holding stocks per trader	1	2.75	2.13	26.3	2.01
Number of stocks ever traded	1	22.1	19	96	16.2
Total return per trader (%)	-1069	30.1	3.7	10230	263
B. Social network					
Number of followers per trader	0	8.49	0	2083	70.7
Number of followings per trader	0	10.1	4	294	18.3

This table presents summary statistics of traders’ transactions (Panel A) and social network (Panel B). The “portfolio life” is the number of days between the portfolio’s creation date to its last trade date. The “total return per trader” is a trader’s total return throughout the entire sample period. “Number of followings/ followers per trader” are computed by taking a snapshot of a trader’s network on the day of its last trade and counting the numbers.

Table 1(B) shows a trader’s number of followings and followers. Since the network changes every day, we take a snapshot of a trader’s network on the last day of its portfolio life and only report statistics on that day. It shows that the statistics are exceptionally right skewed: The majority of the traders have only a few followings/followers, except a few having a lot.

## Methods

In this paper, we use two approaches to test the effects of social interaction on the disposition effect. Borrowing from [9, 13], we first split the sample into the periods without and with social interactions. We will then examine the change in the disposition effects between the two periods. Second, without splitting the sample, we will measure the social mechanism continuously and directly by introducing three channels: learning intensity, learning quality, and public scrutinization.

### Splitting samples: The pre-follow and the post-follow periods

**Fig 3:**
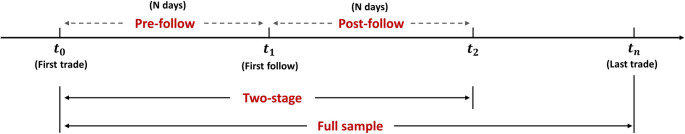
Split the sample into periods before and after social learning.

We observe that a new user usually makes a few trades before starting to follow others’ portfolios. This enables a comparison of trading outcomes under different social interaction settings: the trades made before the first following is less likely to be influenced by peers, while the trades made after are more exposed to social interaction [9, 13]. The impact of social interaction could then be revealed by comparing the disposition effect between the two periods; specifically, we split the sample into two stages: the pre-follow and the post-follow. Fig 3 illustrates the split. Assume a trader made her first transaction on Xueqiu on day *t*_0_ but did not follow any portfolio until *t*_1_. The pre-follow stage is defined as the period from *t*_0_ to *t*_1_; the post-follow stage is defined as the same-length period after she began to follow, that is, *t*_2_ is defined so that *t*_2_−*t*_1_ = *t*_1_−*t*_0_. Period combing both the pre-follow and the post-follow stages (*t*_0_ to *t*_2_) is named the “two-stage” phase. We further require a trader must be present in both stages; otherwise, she will be removed from the sample. The equal length of the two stages helps to reduce the impact of different sample sizes. *t*_*n*_ is the day when the user made her last trade in our sample, and we refer to the period from *t*_0_ to *t*_*n*_ as the “full sample.”

### Measure the social interaction channels

While the above approach is clear to show the impact of social interaction, it treats social interaction as a whole and cannot distinguish different channels of it. Can we measure the channels of social interaction continuously without splitting the sample? To this end, we propose three variables: learning intensity, learning quality, and public scrutinization. Each variable represents a channel of social interaction.

As their names indicate, both learning quality and learning intensity represent the learning activity. They are designed to capture the “active” part of social interaction because learning is an initiative that originates from the user herself. Literature has demonstrated that social learning, loosely defined as copying the winners’ behavior, could help to reduce behavior bias and improve investment return [24]. One intuitive way to measure the learning activity is to count the number of followings. A larger number of followings indicates a larger pool of portfolios that the user is observing, which indicates a stronger motivation to learn. With this reasoning, we first introduce *learning intensity*. Eq (1) defines the *learning intensity* of investor *j* at day *t* as the logarithm of the number of followings at day *t* plus one. *learning intensity*, essentially the out-degree of a node, captures a trader’s willingness to learn.

$${learning\;intensity}_{jt}=\ln\left(number\;of\;followings_{jt}+1\right)$$ (1)

A significant shortcoming of *learning intensity* is that it cannot capture the content of learning. Since learning is a process of information acquiring, we try to capture the content of learning by measuring the information advantage acquired. [7] proposes that the information advantage of a trader could be proxied by her centrality score in the network. In social network analysis studies, centrality measures a node’s closeness to the center of a network. A high centrality score indicates that a node is deeply and centrally embedded in the network [25–28].

Centrality score comes with many variants. This paper uses PageRank centrality [19] to capture a trader’s information advantage in the network. PageRank was initially developed to reflect the importance of websites [24], and in our setting it provides a more elaborate technique to determine the information advantage of a trader than merely counting the number of followings or followers. To understand what centrality implies, suppose every trader in the network starts with the same score or importance. Whenever a trader follows someone in the network, she implicitly votes for him and passes her score to him. This voting game is played repeatedly. At last, someone in the network would emerge with the highest score or importance, because other significant users follow him. From economics’ perspective, these people are centrally positioned in the network and hold an informational advantage [7].

Equipped with the concept of centrality, we move on to develop a measure to capture a trader’s information advantage through learning. Given trader *j* and her N followings (out-going neighbors), we first compute the centrality score for each of her followings at day *t*, *centrality*_*njt*_. These scores tell if the trader is learning from the important ones. We then average these scores to produce a single metric, *learning quality*, as in Eq (2). A larger *learning quality* means a trader is learning from nodes that have an information advantage. We multiply the resulting value by 100 to avoid large regression coefficients.

$${learnign\;quality}_{jt}=\frac{1}{N}\sum_{n=1}^{N}(centrality_{njt}\times 100)$$ (2)

Compared with *learning intensity*, *learning quality* captures the global learning characteristics of a trader. The difference between *learning intensity* and *learning quality* can be shown in the following example. Suppose a trader follows hundreds of portfolios, but every portfolio that she follows has no followers. In other words, she is following unimportant nodes. In such a condition, the investor will end up with a large *learning intensity* but small *learning quality*. To make sure that *learning intensity* and *learning quality* do capture different aspects of learning, we computed the correlation coefficient between them. A Pearson correlation coefficient of 0.12 shows that they are only weakly correlated, supporting the proposed variables’ validity.

Besides *learning intensity* and *learning quality*, literature also suggests public scrutinization, or transparency, as an crucial passive mechanism of social interaction. Public scrutinization refers to the exposure of one’s trading records to others, which is a distinctive feature of social trading platforms. Public scrutinization could impact the disposition effect in multiple ways. [9, 10, 12, 15] associate it with self-image maintaining and a strengthened disposition effect. They find that with the increase of the number of followers, one will become increasingly reluctant to realize losses because she wants to maintain a high reputation to her followers. By contrast, [13, 14] find a negative relationship between public scrutinization and the disposition effect, which they explain as a result of self-consciousness: An increasing number of followers promotes a trader’s awareness of losses and make her adjust the strategy more actively.

Here, we propose using the number of followers (in-degree) as a proxy for passive public scrutiny. Intuitively, a larger number of followers indicates one’s trading records are exposed to more people, which results in stronger public scrutinization [10]. Eq (3) defines *public scrutinization* for trader *j* on day *t*:

$${public\;scrutinization}_{jt}=\ln\left(number\;of\;followers_{jt}+1\right)$$ (3)

## Results and discussion

### Preliminary evidence

According to the definition of the disposition effect, a trader’s disposition effect will increase if her holding period of a gaining position decreases (sell too soon), or her holding period of a losing position increases (hold for too long). In Table 2, we present the average holding period in the pre- and the post-follow stages (with standard deviation in parentheses). We also present the p-value of a t-test under the null hypothesis that the two stages’ holding periods are equal.

**Table 2:** Change in the holding period between the pre-follow and the post-follow periods.

	Pre-follow (1)	Post-follow (2)	P-value^a^ (3)
Average holding period of stocks before sold (days)	15.3 (19.8)	27.2 (39.2)	<0.001
Average holding period of stocks before sold at a gain (days)	14.6 (19.2)	25.1 (37.6)	<0.001
Average holding period of stocks before sold at a loss (days)	15.4 (21.3)	27.1 (42.1)	<0.001

Table 2 presents traders’ average holding period in the pre- and the post-follow stages. Standard deviations are in parentheses.

^a^ P-value is computed under the null hypothesis that the holding periods of the two stages are equal.

Table 2 shows that, in general, traders hold stocks for longer after exposure to social interaction (row 1). When we group the sell trades into those sold at a gain and those sold at a loss, however, a mixed picture is shown. The increased average holding period of stocks before sold at a gain from 14.6 days to 25.1 days (row 2) indicates an increased disposition effect. However, the significantly increased average holding period before sold at a loss from 15.4 days to 27.1 days (row 3) suggests the opposite. Unfortunately, Table 2 cannot tell us the net effect of this asymmetric change in the disposition effect.

**Fig 4:**
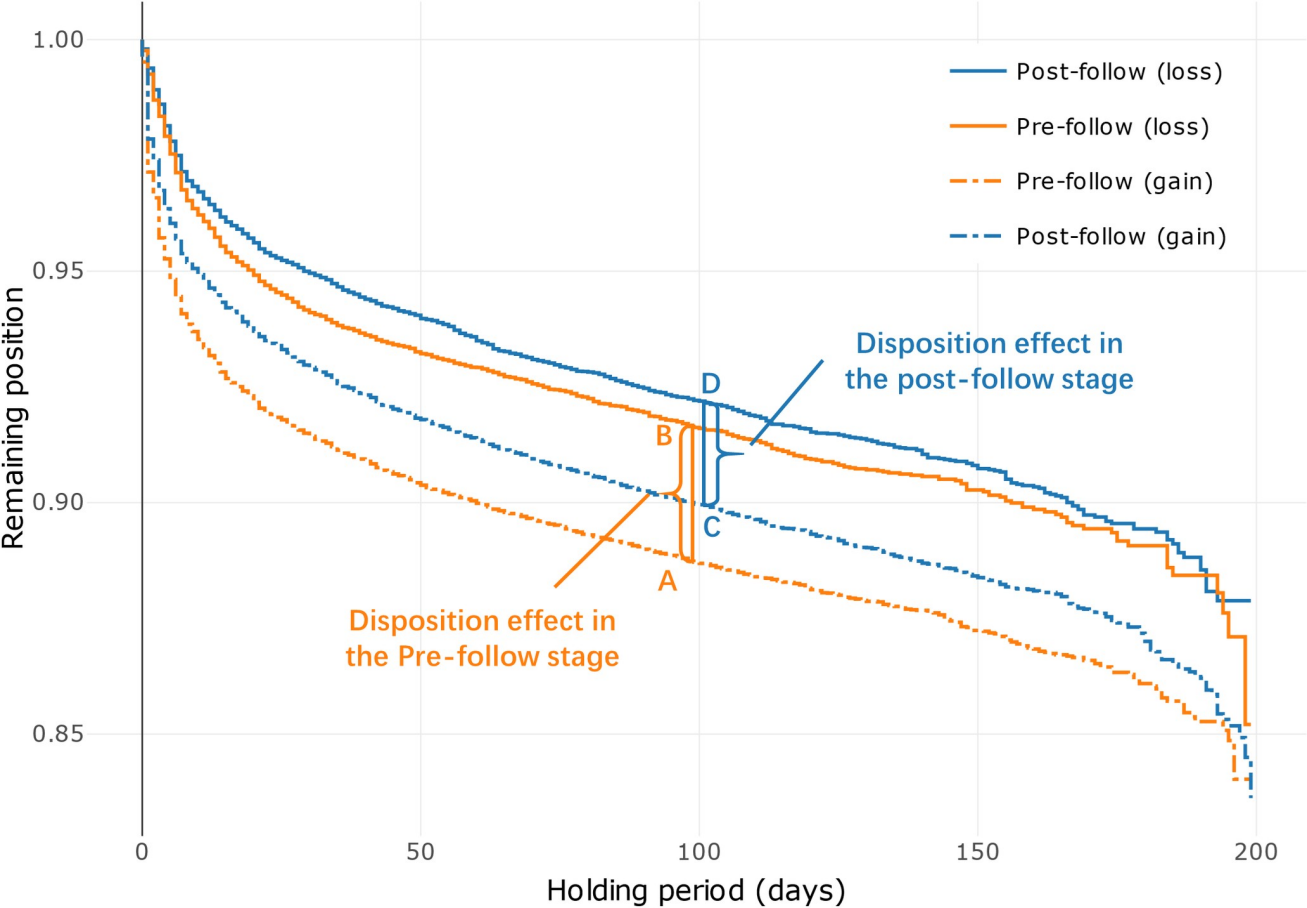
Remaining position vs. holding period. This figure presents the relationship between the remaining position of a stock and its holding period.

To clearly display the net effect, we plot estimates of a Kaplan–Meier survival function [2, 9, 12, 24, 29] in Fig 4, where the outcome of interest is a binary indicator of closing a position. The function shows the remaining position of an unsold stock against the holding period. For example, in Fig 4, a point of a holding period of 100 days and a remaining position of 0.9 means that, on average, 10% of the position is sold within 100 days of purchase. We indicate the pre-follow (post-follow) stage with orange (blue) lines and whether a stock is sold at a gain (loss) with dash (solid) lines, resulting in a total of four lines.

Fig 4 shows three interesting patterns. First, we observe that the solid lines are always above the dash lines within the same stage. This indicates the presence of the disposition effect: Traders are more likely to sell a stock if it is at a gain. Second, the solid and dash lines experience an overall upward shift in the post-follow stage, suggesting that the holding period (regardless of whether the position is at a gain or loss) has increased after exposure to social interaction, confirming Table 2. Third and most important, we observed a reduced gap between the solid and dash line in the post-follow stage, which implies a reduced disposition effect. For example, in Fig 4, point A (B) indicates that at day 100, if a stock is at a gain (loss), 89% (92%) of the position remains unsold. Therefore, the gap (92–89 = 3) measures how more likely a trader is to close the position at a gain than at a loss, which is her disposition effect in the pre-follow stage. Likewise, the gap between points C and D measures her disposition effect during the post-follow stage. It is clear that the gap is narrowed in the post-follow stage, suggesting a reduced disposition effect.

### The change in the disposition effect before and after social interaction

Though Fig 4 successfully shows a negative net change of the disposition effect, it requires one to compute the gap at each holding period day. To produce a single metric of the disposition effect for the entire holding period, we turn to another family of survival function, the Cox proportional hazard regression, to model the sale transactions [2–4, 9].

Eq (4) shows the model. The dependent variable *h*_*ij*_(*t*) measures the hazard rate for stock *j* being sold by investor *i* at day *t* under the influences of factors specified in the right-hand side of the model. *h*_0_(*t*) is the baseline hazard, corresponding to the hazard value with all factor influences being zero. *gain*_*ijt*_ is a binary indicator that equals one if stock *i* is sold at a gain by trader *j* at day *t*, and zero if sold at a loss. A positive coefficient of *gain* suggests the presence of the disposition effect: It means a stock is more likely to be sold if it is a paper gain. Therefore, the change in the disposition effect between the pre- and the post-follow stages could be revealed by comparing the change in the coefficients of *gain*.

$$h_{ij}(t)=h_{0}(t)\exp\left(\beta_{1}gain_{ijt}\right)$$ (4)

Another way to test the disposition effect change between the two stages is to pool the stages together and add a binary period indicator, *post-follow*, which equals one if a trade is made in the post-follow stage. Eq (5) shows the model. The coefficient of the interaction term between *gain* and *post-follow*, *β*_3_, measures the disposition effect change resulting from social learning. A negative *β*_3_ suggests a reduced disposition effect.

$$h_{ij}(t)=h_{0}(t)\exp\left(\beta_{1}gain_{ijt}+\beta_{2}post‐follow_{ijt}+\beta_{3}gain_{ijt}\times post‐follow_{ijt}\right)$$ (5)

Table 3 reports the results. Columns 1–2 are estimated with Eq (4) on the pre- and the post-follow stages separately, while Column 3 is estimated with Eq (5) by pooling the two stages together. In all regressions, we cluster the standard deviations by traders. Table 3 confirms a negative association between social interaction and the disposition effect. Columns 1–2 show that in the pre-follow stage, the hazard ratio for a stock being sold at a gain is 57% (*e*^0.451^−1) higher than that at a loss, while the gap falls to 42.6% (*e*^0.355^−1) in the post follow stage. Column 3 confirms this, where the interaction term between *gain* and *post-follow* shows that the disposition effect has been reduced by 9.06% (*e*^−0.095^−1) in the post-follow stage.

**Table 3:** Social learning and the disposition effect: Cox proportional hazards model.

	Pre-follow (1)	Post-follow (2)	Two-stage (3)
**Gain**	0.451*** (0.015)	0.355*** (0.017)	0.450*** (0.015)
**Post-follow**			-0.127*** (0.020)
**Gain × Post-follow**			-0.095*** (0.023)
**Observations**	316,116	289,584	605,700
R^2^	0.003	0.002	0.003

This table presents the results of the Cox proportional hazards regression specified in Eqs (4) and (5). The Pre-follow stage (Column 1) refers to the period from first entering the platform to first following someone. The Post-follow stage (Column 2) refers to the same length period (see Fig 3) after the first following. Two-stage (Column 3) includes both the pre-follow and the post-follow stages. Standard deviations are in parentheses, and ***, **, and * denote significance at 0.1%, 1%, and 5%, respectively.

As a robustness check, we use Logistic regression to re-estimate the above equations. We first propose Eq (6), where the dependent variable *sale*_*ijt*_ equals one if stock *i* is sold by investor *j* at day *t*, and zero if not sold. The independent variable *gain*_*ijt*_ is a binary indicator for whether stock *i* is sold at a gain by trader *j* at day *t*.

$$logit\left[\frac{P(sale_{ijt})}{1-P(sale_{ijt})}\right]=\beta_{j}gain_{ijt}\;(\mathrm{for}\;\mathrm{every}\;\mathrm{trader}\;j)$$ (6)

**Fig 5:**
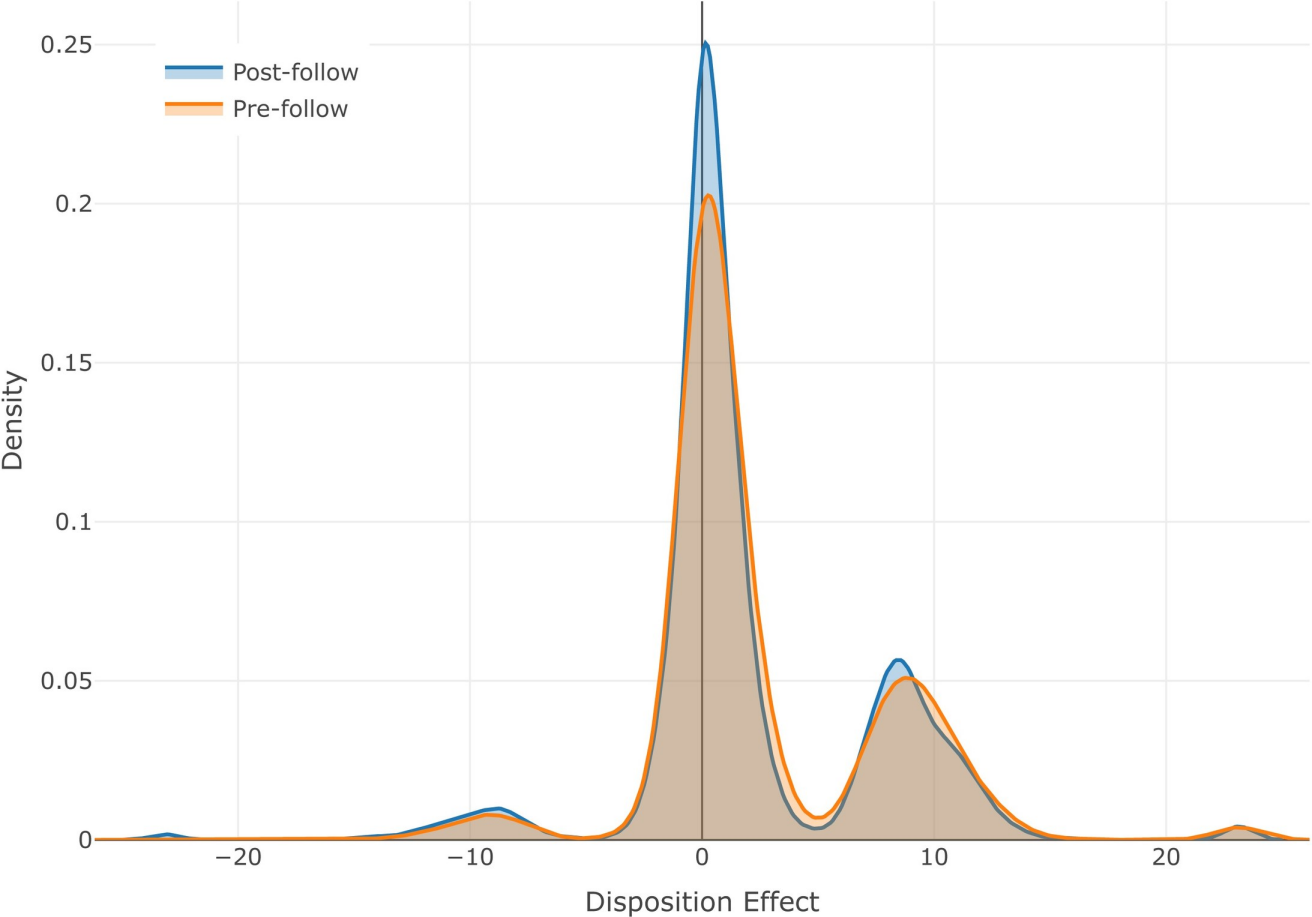
Distribution of traders’ disposition effect before and after social learning. In each of the pre- (orange) and the post-follow (blue) stage, we estimate every trader’s disposition effect with Eq (6) and present its distribution. A positive value in the x-axis indicates the presence of the disposition effect.

To give an intuitive illustration of the change in the disposition effect, we estimate Eq (6) for every trader *j* separately in both the pre- and the post-follow periods. This will give us one *β*_*j*_ (the estimate of the disposition effect) for every trader. We show the distribution of *β*_*j*_ in Fig 5, where the orange (blue) line represents the results from the pre-follow (post-follow) stage. Two notable patterns are observed. First, the distribution of *β*_*j*_ is slightly right skewed, indicating the presence of disposition effect. Second, we find that in the post-follow stage, the magnitude of *β*_*j*_ is reduced: It is more concentrated around zero and less likely to appear in the tails. This pattern is most obvious in the right tail, meaning the magnitude reduction in positive *β*_*j*_s is stronger than that of negative *β*_*j*_s. Since a positive *β*_*j*_ is equivalent to the presence of disposition effect, Fig 5 supports a decrease in the disposition effect.

Eq (7) extends Eq (6) by adding a binary period indicator, *post-follow*, where it equals one if a trade is made in the post-follow stage. The coefficient of the interaction term between *gain* and *post-follow*, *β*_3_, measures the disposition effect change resulting from social learning. *α*_*i*_, *α*_*j*_ and *α*_*t*_ are added to capture the stock, trader and holding period level fixed effects. We also control for the market performance and investors’ trading experience by adding three control variables: momentum (a zero-cost portfolio that is long previous 12-month winners and short previous 12-month loser) [30], active days (number of days since entering the platform), and trade number (number of cumulative trades executed since entering the platform). The three control variables are represented by *controls* in Eq (7).

$$logit\left[\frac{P(sale_{ijt})}{1-P(sale_{ijt})}\right]=\alpha_{i}+\alpha_{j}+\alpha_{t}+\beta_{1}gain_{ijt}+\beta_{2}post‐follow_{ijt}+\beta_{3}gain_{ijt}\times post‐follow_{ijt}+\beta controls+\mu_{ijt}$$ (7)

Table 4 presents the results of Eq (7) and strengthens the evidence that social interaction decreases traders’ disposition effect. Columns 1–2 are estimated on the pre- and the post-follow stage separately without adding the period indicator *post-follow*. We find the disposition effect has decreased from 0.903 in the pre-follow stage to 0.738 in the post-follow stage. Column 3 pools the two stages together and uses *post-follow* to indicate them. Again, the negative coefficient of *gain*×*post-follow* (*β*_3_) supports a reduced disposition effect: If a stock is at a gain, the odds ratio for its being sold is reduced by 10% (*e*^−0.106^−1) after social learning. In Column 4–7, we strengthen the results by adding three control variables (momentum, active days, and trade number) to control for the market performance and the investor experience. Columns 8 and 9 use the full sample for robustness purposes; that is, we include all trades before and after social interaction without requiring the length of these two periods being equal (see Fig 3). All the models give a significantly negative estimate of *β*_3_, implying that enhanced social interaction reduces traders’ susceptibility to the disposition effect.

**Table 4:** Social learning and the disposition effect: Logistic regression.

	Dependent Variable: Sale								
	Pre-follow (1)	Post-follow (2)	Two-stage (3)	Two-stage (4)	Two-stage (5)	Two-stage (6)	Two-stage (7)	Full Sample (8)	Full Sample (9)
**Gain**	0.903*** (0.022)	0.738*** (0.025)	0.814*** (0.020)	0.814*** (0.020)	0.814*** (0.020)	0.814*** (0.020)	0.815*** (0.020)	0.754*** (0.019)	0.754*** (0.019)
**Post-follow**			0.170*** (0.026)	0.169*** (0.026)	0.159*** (0.028)	0.053 (0.028)	0.092** (0.029)	0.084*** (0.020)	0.016 (0.021)
**Gain × Post-follow**			-0.106*** (0.029)	-0.106*** (0.029)	-0.107*** (0.029)	-0.109*** (0.029)	-0.107*** (0.029)	-0.104*** (0.022)	-0.103*** (0.022)
**Momentum**				0.623 (0.873)			0.659 (0.874)		1.060* (0.468)
**Active days**					0.032 (0.037)		-0.355*** (0.049)		-0.094*** (0.021)
**Trade number**						2.775*** (0.282)	4.496*** (0.370)		1.998*** (0.190)
**Trader FE**	Yes	Yes	Yes	Yes	Yes	Yes	Yes	Yes	Yes
**Holding period FE**	Yes	Yes	Yes	Yes	Yes	Yes	Yes	Yes	Yes
**Stock FE**	Yes	Yes	Yes	Yes	Yes	Yes	Yes	Yes	Yes
**Observations**	273,004	253,302	574,726	574,724	574,724	574,724	574,724	1,709,439	1,709,439

This table presents the Logistic regression results of Eq (7). The pre-follow stage refers to the period from first entering the platform to first following someone. The post-follow stage refers to the same length period (see Fig 3) after the first following. Two-stage includes both the pre-follow and the post-follow stages. The full sample includes a trader’s entire trading history. Standard deviations are in parentheses, and ***, **, and * denote significance at 0.1%, 1%, and 5%, respectively.

### The social mechanisms

In the above section, we identified a negative association between social interaction and the disposition effect. In this section, we take a step further by investigating the social mechanisms: the different channels through which social interaction affects the disposition effect. As discussed in the Methods Section (Measure the social interaction channels), we identified three channels: *learning intensity*, *learning quality*, and *public scrutinization*. We add them and their interaction terms with *gain* into Eq (7), resulting in Eq (8). Here a negative *β*_3_, *β*_5_ or *β*_7_ indicates a positive role of *learning intensity*, *learning quality* and *public scrutinization* in reducing the disposition effect.

$$\begin{array}{l} logit\left[\frac{P(sale_{ijt})}{1-P(sale_{ijt})}\right]=\alpha_{i}+\alpha_{j}+\alpha_{t}+\beta_{1}gain_{ijt}\\ \;+\beta_{2}{learning‐intensity}_{jt}+\beta_{3}gain_{ijt}\times {learning‐intensity}_{jt}\\ \;+\beta_{4}{learning‐quality}_{jt}+\beta_{5}gain_{ijt}\times {learning‐quality}_{jt}\\ \;+\beta_{6}public‐scrutinization+\beta_{7}gain_{ijt}\times public‐scrutinization\\ \;+\beta controls+\mu_{ijt} \end{array}$$ (8)

Because *learning intensity*, *learning quality*, and *public scrutinization* are calculated on a daily basis, it allows us to measure the social interaction continuously without the need to split the sample into the pre- and the post-follow periods. Therefore, we estimate Eq (8) on a trader’s entire trading history.

Table 5 presents the results of Eq (8). First, we find that *public scrutinization* is negatively associated with the disposition effect: For one percentage increase in the number of followers, the odds ratio for a paper gain stock being sold decreases by 3.24% (*e*^−0.033^−1, Column 4). This finding supports the claim that public exposure results in more self-consciousness and less disposition effect [13, 14]. This also implies a positive role of social trading platforms: Even if the investor does not build any connection in the network, just by exposing her trading records to others is enough to reduce her behavioral bias.

**Table 5:** Learning intensity, learning quality, public scrutinization, and the disposition effect.

	(1)	(2)	(3)	(4)
**Gain**	0.631*** (0.007)	0.728*** (0.012)	0.633*** (0.008)	0.747*** (0.013)
**Public-scrutinization**	0.009 (0.006)			0.002 (0.006)
**Gain × Public-scrutinization**	-0.041*** (0.005)			-0.033*** (0.005)
**Learning intensity**		0.048*** (0.010)		0.033*** (0.009)
**Gain × Learning intensity**		-0.071*** (0.006)		-0.064*** (0.006)
**Learning quality**			0.637*** (0.080)	0.612*** (0.082)
**Gain × Learning quality**			-0.332*** (0.078)	-0.259** (0.079)
**Momentum**	2.146*** (0.297)	2.122*** (0.297)	2.129*** (0.297)	2.149*** (0.297)
**Active days**	-0.116*** (0.015)	-0.124*** (0.017)	-0.127*** (0.015)	-0.108*** (0.016)
**Trade number**	1.101*** (0.102)	1.114*** (0.102)	1.093*** (0.102)	1.114*** (0.102)
**Trader FE**	Yes	Yes	Yes	Yes
**Holding period FE**	Yes	Yes	Yes	Yes
**Stock FE**	Yes	Yes	Yes	Yes
**Observations**	2,767,176	2,767,176	2,767,176	2,767,176

This table presents the results of Eq (8) with three-channel variables added: *learning intensity*, *learning quality*, and *public scrutinization*. Unlike Table 3 or Table 4, Table 5 does not split the sample into two periods: All the regressions are estimated on a trader’s entire history. Standard deviations are in parentheses, and ***, **, and * denote significance at 0.1%, 1%, and 5%, respectively.

Table 5 also suggests a negative association between *learning intensity* and the disposition effect: For one percentage increase in the number of followings, one’s odds ratio to sell a paper-gain stock decreases by 6.2% (*e*^−0.064^−1, Column 4). To explain this finding, first recall that a high learning intensity means a trader is observing a large pool of portfolios. This pool could facilitate decision making in several ways. [31] demonstrates that when one could choose between social learning (e.g., observing others) and asocial learning (e.g., innovating by trial-and-error) methods to inform a decision, a larger portion of observation compared with innovation could significantly increase the performance. Besides, [31] also suggests that a larger number of options to copy from could increase learning adaptiveness.

We also find that *learning quality* could help to reduce the disposition effect, as indicated by the significantly negative coefficient of *gain*×*learning quality*. Since learning quality represents one’s information advantage acquired through the network, it extends the finding of [7] that information advantage could improve investor return by further indicating that information advantage could also reduce one’s disposition effect.

Why would information advantage have an impact on the disposition effect? The literature on decision making under uncertainty provides us with some insight. Under uncertainty, firms would delay exiting unprofitable projects because they expect the situation will turn better in the future [32, 33]. Similarly, uncertain investors are more likely to delay realizing the losses [34, 35], which would prolong holding losing position and increase the disposition effect. Specifically, the Consequential decision making theory [36] states that in the situation where an investor needs to decide if she should realize the loss or not, she needs to answer the following question: “What are my alternatives (e.g., keep holding the position) and what is the consequence (e.g., the price bounces back and the loss is recovered, or the price keeps dropping and the loss widens) of it?” With less information to support the decision, an investor would hesitate to make decisions, resulting in holding the losing position for an unnecessarily long period of time, and finally, results in an increased disposition effect. By contrast, an investor with information advantage would close a losing position more quickly.

## Conclusion

In this study, we investigate the role of social interaction in reducing investors’ disposition effect. We use a novel data source, the social trading platform, which allows us to collect both investors’ transaction records and social network data. Specifically, we collected data from Xueqiu.com, China’s largest social trading platform, because its directed network largely reduces the “reflection problem.” By splitting the sample into periods before and after the exposure to social interaction, we first show that social interaction helps to decrease the disposition effect. We then investigate the social mechanism by decomposing social interaction into three channels: learning intensity (willingness to learn), learning quality (information advantage through learning), and public scrutinization (exposure of trading outcome to others). We find that all these three channels help to reduce the disposition effect. This is in line with the literature stream that social interactions help investors acquire information advantage and reduce behavioral bias [37–39]. Our paper also points out a positive role in social trading platforms in assisting investors in making better decisions.

There is room for further research to understand better the relationship between social interaction, behavior bias, and asset pricing. For example, whether the decrease of behavior bias (e.g., disposition effect) leads to a higher realized return remains a question. Also, while focusing on retail investors, this study does not investigate social interaction’s implication on the overall market. We expect to address these questions in the future.
